# Supplementary material for: Are carbon emissions trading and green financial instruments synergistic? -Comprehensive quantitative research based on content analysis
Source: PLoS One. 2024 Mar 7;19(3):e0298601. doi: 10.1371/journal.pone.0298601 (PMC10919601; doi:10.1371/journal.pone.0298601)
Supplement: S1 Data — (ZIP) [file pone.0298601.s001.zip › Data/地级市Z_F工作报告.zip!/╜¡╦╒/╦╒╓▌╩╨/└┤╫╘╣1⁄2╓┌║┼í╛┬φ┐╦╩2╛▌═°í┐_2019.docx]

各位代表：

　　现在，我代表苏州市人民政府，向大会报告工作，请予审议，并请市政协各位委员和其他列席人员提出意见。

　　一、2018年工作回顾

　　过去的一年，我们在中共苏州市委的正确领导下，深入学习贯彻习近平新时代中国特色社会主义思想和党的十九大精神，认真落实党中央、国务院和省委、省政府的决策部署，团结依靠全市人民，坚持稳中求进工作总基调，自觉践行新发展理念，按照高质量发展要求，有效应对外部环境深刻变化，全力推进供给侧结构性改革，突出打好三大攻坚战，大力实施十二项“三年行动计划”，全面做好改革发展稳定各项工作，较好完成了市十六届人大二次会议确定的目标任务。

　　1．经济发展稳中有进。预计实现地区生产总值1.86万亿元，增长6.8%。一般公共预算收入首次突破2000亿元，达到2120亿元，增长11.1%。固定资产投资4556亿元，同口径增长4.5%。社会消费品零售总额5747亿元，增长7.4%。工业经济平稳增长，规模以上工业总产值达到3.31万亿元，增长6.1%，工业增加值率提高0.5个百分点。新兴产业产值占规上工业总产值的比重达到52.4%，新一代信息技术、生物医药、纳米技术、人工智能四大先导产业占比达到15.7%。新增国家智能制造新模式和试点示范项目5个，成功举办2018全球人工智能产品应用博览会。工业企业资源集约利用工作取得实效，在全省率先出台差别化用地、用能、信贷政策。顺利完成第四次全国经济普查第一阶段任务。现代服务业优化发展，服务业增加值占地区生产总值比重达到50.8%。实现旅游总收入2609亿元、增长12%，文化产业营业收入增长10%。我市成为首批国家服务型制造示范城市，获评国家级示范企业5家。供给侧结构性改革深入实施，关停淘汰落后低效企业1757家，商品住房库存量去化周期为11个月，新增上市公司8家，降低企业负担360亿元，“补短板”项目完成投资259.7亿元。

　　科技创新成效显著。全社会研究与试验发展经费支出占地区生产总值比重达到2.82%。张家港、常熟、昆山入围国家首批创新型县（市）建设名单。张家港、太仓、相城省级高新区获批设立。启动建设市产业技术研究院、江苏先进材料技术创新中心，与中科院、南京大学、牛津大学等国内外大院大所合作共建重大创新载体26个。高新技术产业产值占规上工业总产值比重达到47.7%。新增国家高新技术企业952家、省级民营科技企业1868家。遴选市级高成长创新型培育企业38家，新增“瞪羚”企业116家。实施人才乐居工程、优秀人才贡献奖励等政策，新增高层次人才2.25万人、高技能人才2.05万人。第十届国际精英创业周、“赢在苏州”系列国际创客大赛成功举办。新增省级众创空间42家。中基协备案股权投资、创业投资机构管理基金规模达到3278亿元，市创新产业发展引导基金设立子基金12个、规模达到220亿元，“科贷通”帮助企业解决贷款356亿元。成立中国青年创新创业板（江苏）区域中心，获批建设中国（苏州）知识产权保护中心。新增国家知识产权示范企业16家。万人有效发明专利拥有量达到53件，比上年增加7件。

　　2．改革开放纵深迈进。成功举办改革开放40周年系列庆祝活动，秦振华同志荣获国家改革先锋称号，苏州工业园区、张家港、昆山经济技术开发区获评江苏改革开放40周年先进集体，6人获评先进个人。政府机构改革有序推进，“放管服”改革持续深化，我市商事制度改革、事中事后监管等政策举措获国务院表彰。新增私营企业9.9万家，注册资本4302亿元。“不见面”审批标准化试点积极开展，全面融入江苏政务服务“一张网”，应上尽上事项达到100%。全市实现“12345”热线分区接听，成立全国首家出入境智慧大厅，在全国率先推广“互联网+不动产抵押登记”模式。综合行政执法改革和经济发达镇改革继续深化，吴江区成立全省首家区级综合行政执法局。财税改革扎实推进，政府债务风险可控。企业征信和金融服务创新富有成效，苏州综合金融服务平台帮助企业解决融资需求累计达到5600亿元。国资国企改革稳步实施，新设混合所有制企业21家，完成企业公司制改革4家，东方盛虹实现重大资产重组。

　　开放型经济发展量稳质升。稳妥应对中美经贸摩擦，大力开拓新兴市场，对外贸易额创历史新高。进出口总额达到3541亿美元，增长12%。获批国家深化服务贸易创新发展试点，服务贸易总额达到246亿美元。实现跨境电商交易额15亿美元，市场采购贸易出口额8.8亿美元。精心做好首届中国国际进口博览会相关工作，我市采购商成交额居全省第一。实际使用外资45.2亿美元，新兴产业和高技术项目占比达到51.5%。完成境外中方协议投资额26.8亿美元。参与“一带一路”建设成效显著，埃塞俄比亚东方工业园二期列入中埃两国政府A类重点支持项目，印度尼西亚吉打邦农林生态产业园被认定为省级境外产业合作集聚区。苏州工业园区深化开放创新综合试验又有11项改革经验在全国推广，昆山深化两岸产业合作试验区在金融开放等方面取得新成效，太仓中德先进制造技术国际创新园正式投运。张家港保税港区获批全省唯一汽车平行进口试点。外贸整体通关时间比上年压缩三分之一，口岸提效降费取得积极成效。首届“一带一路”能源部长会议、中日第五次高级别政治对话、第八届中日韩旅游部长会议、第二十次中日韩环境部长会议等国际会议活动在苏成功举办。出台“惠及台胞55条措施”，两岸经济文化交流合作不断深化。外事、港澳、侨务等工作取得新进展。积极投身长三角一体化，推动道路交通互联互通，加快融入G60科创走廊，嘉昆太协同创新核心圈、青昆吴联动发展等合作机制先后建立。南北挂钩共建、对口支援帮扶积极开展，我市荣获全国东西部扶贫协作先进单位称号。

　　3．城乡建设协调并进。苏州市城市总体规划（2035）和综合交通体系规划形成成果，“多规合一”空间规划信息平台通过验收，完成20个古城街坊城市设计。沪通铁路苏州段一期工程开展铺轨作业，江苏南沿江城际铁路苏州段、通苏嘉甬纳入盐通铁路先行实施段开工建设。加快打通省际断头路，昆山锦淀公路与青浦崧泽大道对接通车，开工建设太仓岳鹿路等连通工程。524国道相城段改扩建、桐泾路北延、独墅湖第二通道以及春申湖路、苏同黎公路快速化改造等一批道路交通项目开工建设。轨道交通三期规划获批，3号线主体完工，5号线加快推进，6号线、S1线开工建设，苏州高新区有轨电车1号线延伸线、2号线开通试运营。苏州港完成货物吞吐量5.32亿吨，太仓港集装箱吞吐量突破500万标箱，太仓港集装箱码头四期、张家港港集装箱码头改扩建项目启动建设。京杭大运河苏州段堤防加固工程全线开工，望虞河除险加固工程提前竣工，阳澄湖中西湖连通工程完工通水。1000千伏淮上线苏通管廊过江隧道全线贯通。新增人防设施130万平方米。5G通信试点稳步推进。生活垃圾分类收集处理加快推广，七子山垃圾焚烧发电提标改造项目取得重大进展。“厕所革命”有序开展，新建改建公共卫生间700座。历史文化名城保护和提升六大工程深入实施，古城区域“扫地扫尾”专项行动取得实效。第四批18个园林进入《苏州园林名录》保护体系，“天堂苏州·园林之城”保护管理工程获亚洲都市景观奖。

　　乡村振兴战略全面实施。农村土地制度、集体产权制度改革不断深化，农民集体收益分配权退出、土地承包经营权有偿退出等国家级改革试点工作取得成效。成功举办首届农民丰收节活动。成立全省首家乡村振兴学堂。全市统一的农村集体资产、资源、资金管理信息系统基本建成，“三资”监管机制创新获评首届“中国廉洁创新奖”。农业现代化稳步发展，推进粮食生产功能区和重要农产品生产保护区划定工作，新增现代农业园区5.38万亩。新型农业经营主体加快培育，组建农业产业化联合体46个，认定新型职业农民1562名。相城区成为全国农村创业创新典型范例。新增国家和省级乡村旅游区5家，实现乡村旅游收入410亿元。美丽镇村建设持续推进，被撤并镇面貌有效改善，新建康居特色村10个、三星级康居乡村350个。常熟蒋巷、吴中西巷和东林渡入选省第二批特色田园乡村试点。提档升级农村公路130公里，改造四五类危桥41座。

　　4．环境治理加快推进。狠抓中央环保督察、省环保督察及“回头看”、长江经济带审计等反馈问题整改，一批突出环境问题得到有效解决。打好污染防治攻坚战，持续开展“两减六治三提升”专项行动，大力实施长江环境大整治环保大提升“百日攻坚”、长江经济带固体废物大排查等系列行动，淘汰低端落后化工企业260家，整治“散乱污”企业（作坊）3.54万家，取缔内河非法码头845个，规模化养殖场污染治理率达到95.9%，完成省下达的煤炭消费总量削减任务。新增危废焚烧年处置能力8.58万吨、工业污泥年处置能力49万吨。水环境治理有效开展，河（湖）长制改革全面落实，在全省最严格水资源管理考核中名列第一。持续推进太湖治理和阳澄湖生态优化行动，4.5万亩太湖围网拆除签约率达到100%。整治黑臭水体525个，中心城区干河清淤26条。国考、省考断面水质优Ⅲ比例分别为68.8%和76%，分别高出考核标准12.5个百分点和28个百分点，通江支流优Ⅲ比例达到95.2%。大气环境治理深入实施，市区空气质量优良天数比例达到73.7%、提高2.2个百分点，PM2.5平均浓度为42微克/立方米、名列全省第二。加强土壤环境保护，完成重点行业4270家企业用地信息采集和风险筛查工作，原苏州溶剂厂北区等地块土壤污染得到治理修复。市区新增及改造绿地350万平方米，全市新增成片造林7520亩，陆地森林覆盖率达到29.9%。常熟成为全球第一批国际湿地城市。太仓、吴江、吴中入选首批省级生态文明示范县（市、区）。

　　5．社会事业与时俱进。推动基础教育均衡优质发展，建成投用69所中小学幼儿园，新增学位6.8万个。全面优化学校内涵建设，推进集团化办学，增加普通高中教育学位5600个。开展教育领域人民群众反映强烈的突出问题专项治理，促进校外培训机构规范有序发展。统筹全市名优教师，打造免费线上教育平台。完善现代职教体系，新增2所国家中等职业教育改革发展示范学校。深入推进名城名校融合发展，支持在苏高校提升办学水平，中国人民大学丝路学院开始招生，昆山杜克大学开展本科阶段教学。加快实施卫生与健康提升行动，持续深化医改，开展全方位全周期健康服务，推动健康惠民。新建和改扩建基层医疗卫生机构24家，建成市第九人民医院，投用市体检中心、苏大附二院高新区医院。新引进高水平临床医学专家团队26个，累计达到53个。张家港、太仓获得世界卫生组织健康城市最佳实践奖。文化事业繁荣兴盛，苏州湾文化中心主体结构封顶，苏州博物馆西馆、吴文化博物馆等项目建设顺利推进，完成671个村（社区）综合性文化服务中心标准化建设。依法加强江南水乡古镇保护，统筹推进大运河文化带建设。成功举办世界遗产城市组织第三届亚太区大会、中国昆剧艺术节、中国苏州评弹艺术节、苏州国际设计周、2018戏曲百戏（昆山）盛典等重大活动。我市荣获全球首个世界遗产典范城市称号。世界记忆项目苏州学术中心正式设立。31部地方志书获评省级优秀成果。启动创建国家全民运动健身模范市，新增健身步道264公里，苏州奥林匹克体育中心、东园体育休闲公园建成投用。成功举办2018冰壶世界杯苏州站、国际田联世界竞走团体锦标赛等重大赛事。我市运动员在第十九届省运会上取得历史最好成绩。

　　社会大局和谐有序。“文明苏州·德善之城”建设不断深化，13人获选中国好人，9户家庭被评为全国和省五好家庭。城乡社区治理现代化全面推进，全科社工模式得到推广。我市成为首批国家守信激励创新试点城市，荣获全国守信激励创新奖。建成市社会综合治理网格化联动大数据中心，实战效能和预测预警功能不断增强。严格落实安全生产责任制，强化安全生产监管，事故起数、死亡人数分别下降11.4%和14.6%。开展“331”整治火灾隐患专项行动，火灾起数、死亡人数分别下降29.9%和36.4%。妥善处置群众信访诉求，有效化解一批矛盾纠纷。建立姑苏动态巡防体系，启动“城市盾牌”智慧安防建设，扫黑除恶专项斗争开局良好，完成首届中国国际进口博览会等重大活动苏州安保任务，社会平安稳定局面进一步巩固。民族宗教工作全面加强。深化军民融合发展，大力支持军队改革和部队战斗力建设，连续2届实现省级双拥模范城“满堂红”。

　　6．民生福祉持续增进。城镇、农村居民人均可支配收入分别达到6.35万元和3.24万元，增长8%和8.2%。全力办好民生实事，群众关心的热点难点问题得到进一步解决。全市新增就业17.6万人，扶持成功创业1.9万人，苏州籍应届高校毕业生就业率达到98.7%，城镇登记失业率为1.78%。大病保险制度全面实施，长期护理保险试点范围继续扩大。城乡居民基础养老金和医保待遇稳步提高，最低生活保障标准达到每人每月945元。居家和社区养老服务改革、医养融合试点工作持续推进，建成开业区域性养老服务中心4个，新增养老机构床位1054张，其中护理型床位878张，新建日间照料中心110家。精准扶贫帮困力度不断加大，在全省率先建立因病致贫家庭生活救助长效机制。老龄、妇女、青少年、残疾人、红十字、关心下一代等事业取得新成效，我市在中国城市公益慈善指数排名中名列前茅。保障性安居工程开工建设2.1万套。新增缴存住房公积金职工81.6万人，缴存覆盖面提高3.6个百分点。市区新辟、优化公交线路63条，古城区实现新能源公交车全覆盖。全市新增公共自行车站点180个。我市成为国家公交都市建设示范市，获得第29届智能交通世界大会承办权。开工建设市现代农产品物流园，高质量完成地方粮食储备任务，吴中区获评全国首个放心粮油供应网络建设示范县（区）。食品药品安全放心行动扎实开展，食用农产品区域性风险防控体系基本建成。市区居民消费价格总水平涨幅为2.6%，市场物价总体稳定。

　　我们切实加强政府自身建设，努力提升服务效能。深入推进“两学一做”学习教育常态化制度化，始终在思想上政治上行动上同以习近平同志为核心的党中央保持高度一致。认真开展解放思想大讨论活动，持续深化“六个一”基层走访调研，进一步改进工作作风，不断巩固与人民群众的紧密联系。严格执行中央八项规定实施细则和省、市实施办法，深入整治“四风”问题新表现，集中整治形式主义、官僚主义，严肃查处群众身边的不正之风。厉行勤俭节约，“三公”经费继续下降。狠抓重点领域反腐倡廉工作，政府系统党风廉政建设全面加强。坚持依法行政，我市在中国法治政府评估中位列地级市第一位。我市实施“四大工程”、扎实推进高水平开放经验获国务院大督查通报表扬。加强审计监督，重大政策措施跟踪审计、领导干部经济责任和自然资源资产离任审计、重点投资项目和国企国资审计成效明显。自觉接受市人民代表大会及其常委会的法律监督、工作监督和市政协的民主监督，坚持重大事项向市人大常委会报告、向市政协通报，注重发挥各民主党派、工商联、无党派人士和人民团体的参政议政作用。组织实施人大议案1件，办复人大代表建议267件、政协提案428件。

　　各位代表，过去的一年，我们面对复杂多变的形势，团结拼搏、砥砺奋进，取得了来之不易的成绩。这是中共苏州市委坚强领导的结果，是全市人民不懈奋斗的成果。在此，我代表苏州市人民政府，向全市人民，向各位人大代表、政协委员，向各民主党派、工商联、无党派人士，向各人民团体、各界人士，向驻苏人民解放军和武警官兵，表示崇高的敬意！向关心和支持苏州建设的海内外朋友，表示衷心的感谢！

　　在看到成绩的同时，我们也清醒地认识到经济社会发展和政府工作仍然存在不少问题，主要是：经济下行压力加大，产业转型任重道远，自主创新能力和经济发展质量需要进一步提高；文化、教育、卫生、养老、交通等方面的供给与人民群众期盼仍有差距；节能减排、污染防治、安全生产等任务紧迫而艰巨；转变政府职能、创新社会治理、加强城市管理、维护社会公平、增进民生福祉还需下大力气。对于这些问题，必须采取更为有效措施，努力加以解决。

　　二、2019年主要任务

　　2019年是新中国成立七十周年，是决胜高水平全面建成小康社会关键之年，做好今年工作意义重大。当前，世界面临着百年未有之大变局，外部环境复杂严峻，宏观形势稳中有变、变中有忧，我市经济运行不确定不稳定因素也明显增多。面对挑战，我们更应看到，我国经济长期向好的基本面没有改变，改革开放40年的发展为苏州克难而进、开创美好未来打下了坚实基础。我们既要正视困难、增强忧患意识，更要坚定信心、保持战略定力，紧紧抓住国家战略叠加带来的重大机遇，顺势而为、乘势而上，奋力拓展新空间、增创新优势、实现新飞跃，推动高质量发展走在时代最前列。

　　根据中共苏州市委十二届七次全会的部署，今年政府工作的总体要求是：以习近平新时代中国特色社会主义思想为指导，全面贯彻党的十九大和十九届二中、三中全会精神，对标总书记对江苏工作的重要指示，按照党中央、国务院和省委、省政府决策部署，坚持稳中求进工作总基调，坚持新发展理念，坚持推动高质量发展，坚持以供给侧结构性改革为主线，坚持深化市场化改革、扩大高水平开放，加快建设现代化经济体系，继续打好三大攻坚战，认真做好“六个稳”工作，持续实施十二项“三年行动计划”，统筹推进稳增长、促改革、调结构、惠民生、防风险工作，保持经济持续健康发展和社会大局稳定，为高水平全面建成小康社会收官打下决定性基础。

　　全市经济社会发展的主要预期目标是：地区生产总值增长6.5%左右，一般公共预算收入增长6.5%，固定资产投资保持稳定，社会消费品零售总额增长7%左右，进出口总额总体稳定，全社会研究与试验发展经费支出占地区生产总值的比重为2.87%左右，企业研究与试验发展经费投入增长10%左右，万人有效发明专利拥有量56件，全员劳动生产率增长6.5%左右，工业增加值率提高0.4个百分点左右，居民人均可支配收入增长高于地区生产总值增幅，居民消费价格总水平涨幅控制目标为3%左右，城镇登记失业率控制在3%以内，单位地区生产总值能耗下降、主要污染物排放量削减完成省下达任务。

　　新一年重点做好以下八个方面工作：

　　1．聚焦提质增效，全力保障经济稳健运行。稳字当头，多措并举增投资、扩消费、稳外贸，不断增强内生动力，促进经济平稳健康发展。一是积极增加有效投入。加快建设总投资超过1.1万亿元的228项市级重点项目，年内完成投资超过1400亿元。保持工业投入稳定，新兴产业投资占工业投资比重达到70%。实施总投资超千亿元的100个重点工业投资项目，着力推进中科曙光安全可控信息技术产业化基地、长城宝马光束汽车、京东方智慧终端等单项投资百亿元以上的产业项目。精准聚焦公共服务和基础设施等领域，加快实施一批“补短板”项目。二是持续推动消费升级。完善促进消费体制机制，扩大文化教育、旅游休闲、医疗健康、养老家政等服务消费，培育新的消费增长点，进一步激发居民消费潜力。加大对农村电商、平台电商等新零售的扶持力度，推动传统商贸创新发展。三是提质发展对外贸易。适应新形势、把握新特点，推动出口市场多元化，稳定进出口贸易。深入推进加工贸易转型升级和综合保税区高水平开放，引导加工贸易向内外协作配套、两个市场并举转型，向高附加值环节延伸。推进外贸企业自主品牌建设，提高一般贸易比重。做好国家深化服务贸易创新发展试点工作，服务贸易额增长10%。积极扩大进口，精心做好第二届中国国际进口博览会采购组织工作。四是做优做强实体经济。着眼“放水养鱼”、增强后劲，落实“更大规模减税、更明显降费”举措，加强要素保障，强化政策协同，为企业营造更好发展环境。提升金融服务实体经济的水平，完善苏州综合金融服务平台功能，强化股权融资服务，帮助企业融资超过1000亿元。大力支持民营企业发展壮大，切实帮助民营经济解决发展中的困难，让民营经济创新源泉充分涌流、创造活力充分迸发。

　　2．聚焦科技创新，大力提升产业发展层次。坚持以创新引领高质量发展，加快构建自主可控的现代产业体系。一是加强创新载体建设。强化企业创新主体地位，创建国家和省产业创新中心、技术创新中心，加速发展新型研发机构。深化与科研院所、著名高校的合作，吸引更多重大科研机构落户我市。增加研发投入，强化重点领域、关键环节的原始创新，争取突破一批重大原创技术、颠覆性技术、自主可控技术。二是加大人才引育力度。深入实施系列人才计划，重视培养本土人才，积极引进“高精尖缺”人才和团队，新增高层次人才2.2万人、高技能人才2万人，引进创新创业领军人才（团队）500个以上。办好国际精英创业周、国际创客大赛苏州总决赛，加快建设海外人才离岸创新创业基地。三是壮大创新型企业集群。大力实施高成长创新型、“瞪羚”“知识产权登峰”等企业培育计划，健全分级孵化体系，加快打造具有自主知识产权和核心竞争力的创新型企业集群。新增国家高新技术企业1000家、省民营科技企业1000家，高新技术产业产值占规上工业总产值比重提高1个百分点左右。四是优化创新生态系统。深化科技金融结合，扩大“科贷通”和科技保险覆盖面。鼓励创投机构发展，引导更多社会资本设立种子基金、天使基金。加强知识产权保护运用，完善国家技术转移苏南中心等服务平台功能，建成中国（苏州）知识产权保护中心。加快建设苏州国家质量基础基地。

　　以高端化为导向，推动先进制造业和现代服务业深度融合，加快产业转型升级。一是大力发展先进制造业。集中力量打造先导产业创新集聚区，实现四大先导产业产值7000亿元以上。培育发展高端装备、汽车及零部件、集成电路等10个千亿级新兴产业集群，新兴产业产值占规上工业总产值比重提高1个百分点。二是优化发展现代服务业。着力发展金融、商务、研发、信息等生产性服务业，积极培育知识产权、人力资源、检验检测等新兴服务业，争创省级生产性服务业集聚示范区3家。加快发展数字经济、总部经济、平台经济、共享经济等业态模式，新增服务业创新型示范企业35家、总部企业20家，数字经济突破2000亿元。加快文旅融合，推动“旅游+多产业”发展，完善苏州旅游总入口功能，强化旅游市场秩序长效管理，做优古城全域旅游核心区，高质量推进旅游风情小镇创建工作。三是改造提升传统产业。持续开展“千企技改升级”行动，技改投资占工业投资比重保持在70%左右。深入实施智能制造“十百千万”工程，新增智能工厂10家、示范智能车间100家，推广工业机器人超过5000台。推进国家服务型制造示范城市建设，深化制造业与互联网融合发展试点，创建省级重点工业互联网平台3家、工业互联网标杆企业50家。完成第四次全国经济普查登记工作。用好工业企业资源集约利用平台，全面落实资源要素差别化政策。坚决淘汰低端落后及过剩产能，确保完成省下达任务。

　　3．聚焦改革开放，持续增强发展动力活力。突出重点领域和关键环节，以更大决心、更大勇气、更大力度把改革开放推向深入。一是深化“放管服”改革。加强“不见面”审批规范化标准化建设，推进政务服务“一网通办”和“一号通答”，最大限度利企便民。深入实施“多证合一”改革，加快实现市场主体“一照一码走天下”。做好第二批经济发达镇行政管理体制改革试点工作。二是深化财税金融改革。调整理顺市、区财政事权和支出责任，健全基本公共服务财力保障机制。落实国税地税征管体制改革任务，有序推进社保费和非税收入征管职责划转工作。优化地方金融监管体制，争创首个国家级小微企业数字征信试验区。争取设立昆山金融综合改革试验区。三是深化国资国企改革。坚持政企分开、政资分开和公平竞争原则，做强做优做大国有资本。加快股权多元化和混合所有制改革，完善法人治理结构，提高国有资本配置效率。推动国企聚焦主业，压缩管理层级，实现“瘦身健体”、提质增效。

　　深化开放合作，推动形成全面开放新格局。一是建设更高水平的开放平台。更高标准推进开发区改革创新，率先探索“大部制”、市场化招商等改革。以纪念苏州工业园区开发建设25周年为契机，进一步拓展开放创新综合试验。提高昆山深化两岸产业合作试验区、中德中小企业合作区建设水平，启动苏州（相城）中日智能制造创新产业园建设。加快复制推广自贸试验区改革试点经验，争取设立国家自贸试验区。深入推进跨境电子商务等试验试点工作，深化国际贸易“单一窗口”和全国通关一体化改革。二是推进“一带一路”交汇点建设。加强与“一带一路”沿线国家和地区的经贸合作，扩大贸易规模。支持“走出去”发展，引导企业抱团建设境外经贸合作区。推动中欧（苏州）班列持续发展，加快建设江苏（苏州）国际铁路物流中心。三是提升双向开放水平。全面落实市场准入负面清单，积极吸引外商投资，提高使用外资质量，新设外资企业地区总部30家。鼓励本土企业跨国经营，拓展市场渠道，开展境外研发，打造国际品牌。做好外事、对台、港澳和侨务工作，促进经贸合作与人文交流。抓好挂钩共建、对口支援和协作帮扶工作。

　　4．聚焦区域一体，着力提升城市发展能级。积极主动融入长三角世界级城市群建设，努力打造独具魅力的国际化大都市。一是以大局意识融入一体发展。紧紧抓住长三角区域一体化发展上升为国家战略的重大机遇，对接落实长三角发展规划和三年行动计划，协调市域各板块发展，加强与上海及周边城市的全方位合作，推动区域协同创新、特色产业协作共赢、基础设施互联互通、环境治理联防联控、民生工程共建共享。二是以超前眼光规划城市布局。统筹各类空间性规划，加快建立规范有序的空间规划体系，高质量编制国土空间总体规划（2035）。认真开展全市第三次国土调查。加快建设“多规合一”信息平台，促进“多规融合”。完成国家城市设计试点任务，实现古城城市设计全覆盖。开展规划建设苏州机场前期工作。三是以工匠精神推进城市建设。精心策划，精细施工，高标准完善基础设施，充分彰显苏州精致典雅特质。加快实施沪通铁路一期、江苏南沿江城际铁路苏州段工程，建设太仓港港口支线铁路，力争开工沪苏湖、通苏嘉甬铁路。扎实推进桐泾路北延、苏同黎公路快速化改造、312国道苏州东段和524国道相城段改扩建。建成轨道交通3号线，抓紧建设5号线、6号线、S1线，开工建设7号线、8号线。推动沿沪、沿浙地区断头路建设，加强与上海的轨道交通对接。加快推进生态修复、城市修补国家试点工作，持续开展海绵城市和地下综合管廊试点项目建设。启动白鹤滩—苏州特高压输变电工程，投运1000千伏淮上线苏通管廊。新增人防设施100万平方米。推动“光网”建设，做好5G通信试商用工作。实施“厕所革命”新三年行动计划，年内改造提升公共卫生间150座。四是以“绣花”功夫抓好城市管理。加强历史文化名城保护，加快推进平江片区整体保护示范工程，继续实施虎丘地区综合改造、桃花坞片区综合整治和保护利用等重大项目。开展建成区违法建设专项治理，持续推进城中村、老旧区域环境综合整治，深入开展户外广告设施整治、市容街景提升等专项行动，完成中心城区20条主次干道及60条支路街巷架空线整治工作，进一步靓化城市颜值。深入推进道路交通系统治理，整体提升“保畅通、保安全”水平。

　　5．聚焦乡村振兴，深入推动城乡融合发展。坚持农业农村优先发展，进一步提升城乡一体化水平。一是优化完善乡村规划。加强乡村规划建设管理，统筹安排村庄和产业布局。深入推进“三优三保”落地见效，有效保护“四个百万亩”，分批编制村庄建设详规。二是加快建设现代农业。发展高效设施农业，推进农田、池塘标准化改造，新增现代农业园区4万亩。扶持农业龙头企业发展，创建一批“苏”字头农产品区域公用品牌。培育农民合作社、家庭农场等新型经营主体，新增农业产业化联合体20个，新认定新型职业农民1200名。大力发展共享农庄、乡村民宿，促进农文旅深度融合。三是持续深化农村改革。深入推进土地承包经营权有偿退出等国家改革试点，力争早日通过验收。落实农村土地“三权分置”制度，全面完成确权登记颁证工作。深化农村集体产权制度改革，探索推行股权继承、转让、抵押和担保。发挥乡村振兴投资基金杠杆效应，撬动更多社会和金融资本投向农业农村。四是发展壮大集体经济。创新农村集体资产运营方式，鼓励村级集体经济联合抱团异地发展，促进集体资产保值增值。加强农村集体“三资”监管，实现农村综合信息管理服务“一网通”。继续做好经济薄弱村精准帮扶转化工作，支持薄弱村建设富民载体项目。五是扎实开展农村人居环境整治行动。提升农村生活垃圾和污水治理水平，实施农村黑臭水体整治，规范农村住房建设管理。持续推进美丽镇村建设，全面开展第二批19个被撤并镇的镇区整治提升工作，建好省级特色田园乡村试点和市级试点村庄，建成350个三星级康居乡村和10个康居特色村。创建省“四好农村路”示范市，改造农村公路46.3公里。

　　6．聚焦污染防治，加快建设美丽宜居家园。深入践行“绿水青山就是金山银山”理念，以“共抓大保护、不搞大开发”为导向，扎实开展“两减六治三提升”专项行动，坚决打好污染防治攻坚战。一是着力解决环境突出问题。继续狠抓中央、省环保督察及“回头看”等反馈问题整改，按期完成年度整改任务。持续开展长江环境大整治环保大提升行动，编制长江岸线资源保护利用和开发规划，全面加强长江生态保护与修复。深化河（湖）长制和断面长制改革，开展大江大湖及入江入湖支流水岸同治攻坚行动，推进新一轮东太湖整治，全面完成太湖养殖围网拆除工作，实施阳澄湖生态优化、城区清水等工程，推动河湖水质持续向好。扎实推进重点行业提标改造、燃煤锅炉整治、挥发性有机物治理等重点举措，确保优良天数比例和PM2.5平均浓度达到省考核要求。建成土壤污染防治管理信息平台，强化污染场地风险管控和治理修复。二是大力提升环保处置能力。推进市区生活垃圾处置系列工程，完成七子山垃圾焚烧发电改造一期项目建设。推广生活垃圾分类处置，城乡生活垃圾分类设施覆盖率达到80%。加强城乡生活污水处理设施建设，新增污水管网400公里、日处理能力20万吨。开展河道淤泥综合处置及资源化利用试点，建设工业污泥和固废处置项目，新增危废焚烧年处置能力7.8万吨。三是全力筑牢生态安全屏障。严格落实生态保护红线、环境质量底线、资源利用上线和生态环境准入清单，滚动实施生态文明建设重点工程。加强耕地质量监测，推行耕地轮作休耕。深入推进“绿色苏州”建设，新增森林抚育面积4万亩，市区新增及改造绿地350万平方米。加强湿地公园建设和生态保护修复，探索建设生态涵养发展实验区。四是强力推进环境监管执法。完成环保机构垂直管理改革，完善环境监测网络，提升监测能力。健全以排污许可证为核心的“一证式”环境监管体系，建成打赢污染防治攻坚战协同推进平台二期工程。深入开展重点行业企业环保专项执法行动，严厉打击各类环境违法行为。

　　7．聚焦富民惠民，全面提高人民生活质量。瞄准高水平全面建成小康社会的硬任务，精心做好各项民生工作，不断满足人民日益增长的美好生活需要。一是更高质量推进就业创业。把稳就业摆在突出位置，统筹抓好就业困难人员的就业帮扶，确保全年新增就业15万人，提供应届高校毕业生就业岗位10万个，苏州籍高校毕业生就业率保持在95%以上。持续实施创业促进系列行动计划，大力推进众创空间等创新创业载体建设，新增省级创业型园区2家。二是织密扎牢社会保障网。针对中小微企业、灵活就业人员、新就业形态人员等重点群体，实施精准扩面，促进全民参保。实现职工基本养老保险、基本医疗保险市区统筹，不断拓展大病保险、长期护理保险试点的政策惠及面。持续深化医疗服务价格机制改革，建立药品耗材集中采购使用监管系统。坚决打好精准脱贫攻坚战，加大对因病因灾致贫等困难群体帮扶救助力度。发展健康养老服务，启动10个区域性养老服务中心建设，新建日间照料中心100家、养老机构床位1000张。加快公共场所母乳哺育设施建设，新增母乳哺育室67个。推广残疾人“幸福亮居”工程，帮助更多残疾人改善家居生活环境。促进公益慈善事业健康发展，发挥红十字会人道救助作用。三是积极回应民生关切。深化食品药品安全放心行动，创建食品安全示范城市。加强粮食安全保供，推进农贸市场标准化建设和管理。构建长效机制，促进房地产市场平稳健康发展。进一步扩大住房公积金制度覆盖面，增加保障性住房供给。市区新辟、调整公交线路30条，实现内环高架内新能源公交车全覆盖。强化市场价格监测分析与应急响应，确保价格总水平基本稳定。

　　优化提升基本公共服务，提高市民群众“隐性收入”。一是着力办好人民满意的教育。新建、改扩建中小学幼儿园37所，新增学位4.8万个。深入推进中小学集团化办学，扩大优质普通高中资源覆盖面。全面推行义务教育阶段课后服务，提升线上教育的针对性和实效性，推动校外培训机构长效治理。办好全国第六届中小学生艺术展演活动。实施职教卓越提升行动，持续完善现代职教体系。推动在苏高校发展，支持西交利物浦大学太仓校区、西北工业大学太仓校区建设，积极筹建市属应用型本科大学。二是扎实推进健康中国典范城市建设。全面落实健康苏州系列行动计划，构建集医疗、康复、保健等一体的卫生服务体系。健全分级诊疗制度，创新发展紧密型医疗联合体。完善全科医生培养与使用激励机制，壮大全科医生队伍。启动建设市妇幼保健院、市儿童健康发展中心、市转化医学中心和太湖新城医院等项目，加快建设苏大附一院二期、市中医院二期项目和市独墅湖医院、市疾控中心，新建、改扩建20家基层医疗卫生机构。实施体育惠民行动，新增健身步道270公里，建成市运河体育主题公园和江苏省苏州体育训练基地，筹建苏州太湖足球运动中心。发展体育休闲产业，办好苏州（太湖）马拉松等赛事活动。三是大力促进文化繁荣兴盛。创新体制机制，支持文艺院团发展，引导扶持文艺精品创作。深入开展文化惠民系列活动，举办具有江南特质、苏州特色的文化艺术节。加快建设苏州湾文化中心、狮山文化广场等重点项目，推动基层综合性文化服务中心标准化建设。推进苏州考古博物馆、草鞋山遗址博物馆、桃花坞木刻年画博物馆、孙子兵法博物馆等项目，建成投用苏州第二图书馆。更高标准推进文化遗产保护与传承，争创大运河文化带建设国家示范点，完成詹氏花园等修缮保护工程。做好年鉴和地方志书编修工作。出版发行《苏州通史》。

　　8．聚焦安全稳定，切实维护社会和谐有序。把安全发展作为城市现代文明的重要标志，时刻绷紧安全稳定这根弦，为全市人民安居乐业提供坚实保障 。一是持续抓好安全生产。严格落实安全生产责任制，深化重点领域安全生产隐患专项整治，督促企业落实安全生产主体责任，坚决防范遏制重特大安全事故。巩固深化“331”整治火灾隐患专项行动成果，着力实现从刚性治标向长效治本转变。二是全力保障公共安全。完善社会综合治理网格化联动机制，优化社会治安防控体系和全市域动态巡防体系，扎实开展智慧警务建设。依法严厉打击各类违法犯罪，纵深推进扫黑除恶专项斗争，坚决破除黑恶势力“关系网”“保护伞”。建立健全应急管理长效机制，提高应急处置实战化水平。切实履行地方金融监管责任，严厉整治非法金融活动，认真落实地方隐性债务化解方案，坚决打好防范化解重大风险攻坚战。三是全面加强社会治理。培育践行社会主义核心价值观，扎实推进文明城市长效管理，确保年度测评位列全国第一方阵。大力开展新时代文明实践中心建设，倡导推广好人榜、孝子榜、慈善榜，传承弘扬文明风尚。深入实施“七五”普法规划，不断健全公共法律服务体系。加强社会诚信建设，推动各县级市、区争创国家级信用示范县（区）。持续深化全科社工、社区服务社会化等改革，争创国家和省级社区治理服务实验区。常态开展信访积案化解攻坚工作，强化各类调解的衔接联动，提升源头化解矛盾纠纷能力。做好民族团结进步和宗教工作。推动军民融合深度发展，积极参与创建苏南国家军民融合创新示范区。完善国防动员体系建设，搭建兵役工作一站式服务平台。广泛开展拥军优属活动，落实优抚安置政策，建立覆盖市县镇村各级的退役军人服务体系，在全社会形成关心国防、支持军队、尊重军人的良好风尚。

　　三、全面加强政府自身建设

　　实现高质量发展，需要高效率的机制、高水平的服务和高素质的队伍。我们必须持之以恒强化政府自身建设，坚持党的领导，加强政府系统党的建设，牢固树立“四个意识”，始终坚定“四个自信”，坚决做到“两个维护”，全面落实中央、省和市委重大决策部署，高标准开展“不忘初心、牢记使命”主题教育，加快转变政府职能，不断提高行政效能，更好地为全市人民服务。

　　1．着力打造法治政府。坚持依宪施政、依法行政，推动法治成为苏州核心竞争力的重要标志。尊崇宪法权威。认真学习宪法，准确把握宪法的核心要义和精神实质，切实把宪法原则、宪法精神、宪法使命贯彻到政府工作的全领域、各环节。坚持依法行政。严格按照法定权限用权，切实做到手中有权当尽责，心中有规不逾矩。坚决贯彻市人民代表大会及其常委会决议决定，认真落实协商民主有关规定，不断提高人大议案、代表建议、政协提案办理质量和满意率。开展重大行政决策规范管理标准化体系化试点，充分发挥新型智库作用，促进政府决策科学化民主化法治化。加强执法队伍建设，严格规范公正文明执法。做好行政复议案件办理、行政诉讼案件出庭应诉等工作。主动接受监督。依法接受人民代表大会及其常委会的法律监督和工作监督，自觉接受人民政协的民主监督，接受监察监督、司法监督、审计监督、社会监督和舆论监督。加强政府制度建设，完善政府内部层级监督，进一步扎紧制度笼子，让权力在阳光下运行。深化政务公开，强化晒权督权，保障人民群众的知情权、参与权和监督权。

　　2．着力打造创新政府。唱响创新“主旋律”，激发信仰、信念、信心的精神力量，不断增强政府工作的开拓性和创造性，争做努力奔跑的追梦人。大力弘扬创新精神。进一步解放思想，大胆探索，不断丰富“张家港精神”“昆山之路”“园区经验”的时代内涵。认真落实鼓励激励、容错纠错、能上能下“三项机制”，积极宣传改革创新、干事创业的先进典型，旗帜鲜明地支持改革者、鼓励创新者、宽容实干者。不断改进工作方法。按照增强“八个本领”、锤炼“五个过硬”的要求，进一步提高干部适应新时代发展的专业化能力。创新政策供给，有效增进政府政策与市场机制的契合度、与百姓期盼的匹配度。运用大数据提升政府治理现代化水平，推动电子政务转型发展，打造集成化的亲商亲民服务平台。整合城市大数据资源，建设智慧苏州。有效激发机构改革新活力。高标准完成政府机构的改革任务，同步推进县级市、区机构改革，确保改革不拖延、工作不断档、责任不缺位、创新不松懈。科学制定“三定”规定，做到职责明确、职能优化、上下贯通。政府各级各部门要以机构改革为新起点，主动适应新机制，切实履行新职能，充分展现新面貌。

　　3．着力打造高效政府。坚持立说立行、善作善成，不断提升政府工作的效率和水平。勇于担当、真抓实干。始终把工作放在心上、把责任扛在肩上，坚决按照市委决策部署，围绕市人代会确定的年度目标，以时不我待、只争朝夕的劲头抓好任务落实。提振“精气神”，干好“精细活”，扎扎实实做好经济社会发展各项工作。围绕高质量发展开展监测评价，有效发挥考评的导向、激励和约束作用。积极作为、靠前服务。政府系统全体公务人员，在与企业打交道时，要守住底线、把好分寸，竭诚当好服务企业的“店小二”，努力构建亲清新型政商关系。打破形形色色的“卷帘门”“玻璃门”“旋转门”，有效帮助企业爬坡过坎。大力弘扬企业家精神，让企业家在苏州安心投资、成功创业、舒心生活。恪尽职守、一心为民。以造福人民作为政府工作的根本追求，时刻把老百姓的安危冷暖放在心上，对群众关心的事，说一项办一项，项项落实；对群众承诺的事，做一件成一件，件件兑现，不断提高政府执行力、公信力，持续增进人民群众获得感、幸福感、安全感。

　　4．着力打造清廉政府。坚定不移推动全面从严治党向纵深发展，持之以恒正风肃纪。严格落实党风廉政建设责任制。突出抓好中央巡视“回头看”反馈问题整改，坚持举一反三、标本兼治，有效推动党风政风持续改善。认真履行“一岗双责”，把党风廉政建设与政府工作同部署、同安排、同考核。严守党规党纪，把铁的纪律化为政府系统广大党员的日常习惯和行动自觉。切实加强作风建设。坚决贯彻中央八项规定精神和省委、市委各项规定，紧盯“四风”问题新表现，严格落实集中整治形式主义、官僚主义三年行动计划。深入开展基层走访调研活动，听群众诉心声，帮基层解难事，永葆公仆情怀。严控“三公”经费支出，建设节约型机关。倡导开短会、发短文，切实改进文风会风。坚决防止和反对腐败。严厉惩处政府系统滥用职权、贪污贿赂、腐化堕落行为，着力解决群众身边的腐败问题。充分发挥审计的监督作用，推进审计全覆盖，促进权力规范运行。敲响廉洁自律“警世钟”，念好权力运行“紧箍咒”，筑牢反腐拒腐“防火墙”，重点加大对公共资金使用、公共资源交易等领域的反腐力度，严防权力滥用和设租寻租，全力维护风清气正、海晏河清的政治生态。

　　各位代表！使命重在担当，实干铸就辉煌。让我们更加紧密地团结在以习近平同志为核心的党中央周围，在中共苏州市委的坚强领导下，奋力拼搏，开拓进取，为苏州在更高起点上实现高质量发展走在最前列而努力奋斗，以优异成绩迎接中华人民共和国成立七十周年！
